# Does *Astragalus mongholicus* Bunge help promote the healing of wounds? A systematic review and meta-analysis of preclinical animal studies

**DOI:** 10.3389/fphar.2026.1799944

**Published:** 2026-04-17

**Authors:** Jun Zhang, Feifei Zhang, Ruidan Wang, Donghua Yang, Bo Wang, Zhaoyang Zeng, Jinhui Tian

**Affiliations:** 1 Evidence-Based Medicine Center, School of Basic Medical Sciences, Lanzhou University, Lanzhou, Gansu, China; 2 School of Nursing, Gansu University of Chinese Medicine, Lanzhou, Gansu, China; 3 School of Nuclear Science and Technology, Lanzhou University, Lanzhou, Gansu, China; 4 School of Integrative Medicine, Gansu University of Chinese Medicine, Lanzhou, Gansu, China

**Keywords:** *Astragalus mongholicus* Bunge, meta-analysis, preclinical study, systematic review, wound healing

## Abstract

**Background:**

Wounds continue to represent a significant public health challenge globally. In recent years, *Astragalus mongholicus* Bunge, a traditional Chinese botanical drug utilized for wound treatment, has garnered increasing research attention. However, the therapeutic efficacy and safety of this natural botanical drug on wound healing remain poorly understood. This systematic review and meta-analysis aimed to evaluate the efficacy and safety of *Astragalus mongholicus* Bunge in animal wound models to propel future studies towards definitive preclinical and initial clinical trials.

**Methods:**

A systematic search of seven databases was conducted to identify randomized controlled studies that compared *Astragalus mongholicus* Bunge to placebo or a “no treatment” arm in animal models of skin wounds. Two authors independently assessed the risk of bias of the included studies using the Systematic Review Centre for Laboratory Animal Experimentation (SYRCLE) tool for preclinical animal studies and extracted relevant information according to a predesigned extraction form. Random effects models were used to estimate the pooled effect and the data were analyzed using R software.

**Results:**

Finally, 20 citations (including 21 studies) with 559 animals were included. The meta-analysis revealed that compared to the control group, wounds treated with *Astragalus mongholicus* Bunge had a higher wound contraction percentage [standardized mean difference 4.18, 95% confidence interval 2.40–5.96, *P* < 0.0001]. Additionally, the *Astragalus mongholicus* Bunge treatment group exhibited a positive effect in enhancing angiogenesis, facilitating collagen deposition, and diminishing pro-inflammatory factors. Meantime, no harmful events were reported.

**Conclusion:**

*Astragalus mongholicus* Bunge presented positive effects on the process of wound healing in experimental models used, demonstrating the huge potential of this botanical drug for wound treatments. More studies with comparable study protocols should be performed to validate the results of the present systematic review and meta-analysis.

**Systematic review registration:**

This study was registered in PROSPERO (CRD420250641262).

## Introduction

The skin, the largest organ in the human body, serves several functions. The most important is to act as a barrier separating the internal organs of the body from the external environmental threats, including physical and chemical trauma, microorganisms, and radiation, which is essential for maintaining health (Bonifant and Holloway, 2019; Harry, 2025)

However, the skin is often disturbed by various etiologies such as trauma, surgery, burns, and microcirculation dysfunction, leading to acute or chronic wounds (Oliveira et al., 2022; Roshangar et al., 2019). Although it is difficult to provide accurate epidemiological data on skin wounds, a study from the United States reported that wound prevalence increased from 14.5% in 2014 to 16.4% in 2019 (Carter et al., 2023). A published systematic review (SR) that included 11 studies revealed that chronic wounds of various etiologies had a pooled prevalence of 2.21 per 1000 population (Martinengo et al., 2019). It means wounds remain a major public health concern worldwide.

Wound healing is a complex, dynamic process involving hemostasis, inflammation, tissue regeneration, and remodeling (Sorg and Sorg, 2023). Disruption of any of these phases, due to factors including oxygenation, infection, age, sex hormones, stress, diabetes, obesity, medications, alcoholism, smoking, and nutrition, may result in a prolonged healing process or suboptimal recovery (Hom and Davis, 2023; Munoz and Litchford, 2024; Rossboth et al., 2020).

Difficult-to-heal problems of wounds can lead to increased pain, disability, changes in lifestyle, decreased quality of life, psychological problems, and even death (Kapp et al., 2018; Natarajan et al., 2024). Furthermore, they imposed a significant economic burden on both patients and healthcare systems due to long-term medical needs. It is estimated that, only in the USA, 20 billion dollars or more is spent annually on wound treatment (Cruz et al., 2021; Oyama et al., 2020). Hence, how to accelerate wound healing has become a therapeutic challenge of universal concern worldwide.

A correct approach to wound management may effectively influence the clinical outcome. Current standard therapies mainly include surgical debridement, infection management, and regular dressing changes (Oliveira et al., 2022). Recently, due to advances in science and technology, some improved methods for the treatment of wounds emerged, for example, negative pressure therapy and single bioactive factors (Lima et al., 2017; Wong et al., 2024). However, their effect and safety still need to be explored. Moreover, they are expensive. Therefore, the discovery and development of safe, efficient, and affordable ways to encourage wound healing remain a major area of interest for researchers worldwide.

The exploitation of natural resources is one of the most optimized ways to transform cheap raw materials into valuable products for treating human health problems. Botanical drugs are important practices across a range of societies and continue to be used globally as alternative and complementary treatments for a range of disease conditions (Erdemli et al., 2018; Khan et al., 2022; Riaz et al., 2018). *Astragalus mongholicus* Bunge is a traditional Chinese medicine that has been widely used for over two thousand years in the treatment of wounds. More and more modern studies have shown that *Astragalus mongholicus* Bunge and its main active metabolites, usually Astragalus polysaccharides (APS) and Astragaloside IV (AS-IV), may promote wound healing through multiple mechanisms. These include reducing oxidative stress to alleviate tissue damage, as well as modulating the inflammasome process, cell growth and differentiation, and angiogenesis, in addition to improving fibroblast migration and collagen deposition (Cui et al., 2024; Fan et al., 2024; Jia et al., 2024; Zhang Z. et al., 2024).

Despite the positive effects of *Astragalus mongholicus* Bunge exhibited in these wound-healing studies, there is still limited understanding of the effects of this natural botanical drug for wound healing, due to the available evidence being independent, small sample sizes and even inconsistent. In this context, we conducted this systematic review and meta-analysis to evaluate the benefits and safety of *Astragalus mongholicus* Bunge in animal models of wounds in order to completely illustrate the veracity of the conclusions. We believe that this systematic review/meta-analysis of these available preclinical data may be useful for accelerating and optimizing the translation towards clinical investigation.

## Methods

### Protocol and registration

The protocol for this systematic review and meta-analysis was registered prospectively with the International Prospective Register of Systematic Reviews (PROSPERO) (CRD420250641262). The manuscript was prepared in accordance with the Preferred Reporting Items for Systematic Reviews and Meta-Analyses (PRISMA) 2020 checklist to ensure transparent and comprehensive reporting (Page et al., 2021) (Supplementary Table S1).

### Eligibility criteria

We employed the widely recognized PICOS framework to define the study eligibility criteria. This standardized methodology enables a rigorous and unbiased process for identifying, selecting, and evaluating relevant studies, which is crucial for ensuring the objectivity and reproducibility of our analysis. The specific inclusion and exclusion criteria for each PICOS element are detailed below.

#### Population

All *in vivo* animal models of skin wounds were included, with no restrictions regarding age or gender. We excluded studies involving only *in vitro* or non-mammalian species (such as fish).

#### Intervention

The intervention was defined as *Astragalus mongholicus* Bunge, including its two primary bioactive metabolites (APS or AS-IV), whether they are used alone or incorporated into novel materials. There were no restrictions on dosage form, concentration, frequency of administration, dose, intensity, or duration of intervention employed.

#### Comparator

The control group was administered with nonfunctional solutions such as PBS or normal saline, or no treatment. Studies with missing experimental or control groups were excluded.

#### Outcomes

The primary outcome of this study was wound healing, such as the wound contraction percentage (change since baseline) and time to complete healing (days/months to closure). Secondary outcomes included assessments of angiogenesis, collagen deposition, inflammatory cytokine levels, and adverse events. The reporting of primary outcomes was mandatory for inclusion; consequently, studies lacking primary outcome data were excluded.

#### Study design

All included studies were randomized controlled studies.

Studies were excluded from this systematic review and meta-analysis when they adhered to the following criteria: (1) articles were not published in the English or Chinese language; (2) the studies presented duplicate or redundant data; (3) the studies were reviews, correspondence letters, case reports, comments, *etc.*

### Information sources and search strategy

A systematic search was conducted across multiple databases, including PubMed, Embase (*via* the Embase.com platform), Science Citation Index Expanded (*via* Web of Science), EBSCO, Chinese Biomedical Literature Database (CBM), Chinese Digital Journals Full-text Database (CNKI), and Wanfang database, from their inception to June 2025 with no language restrictions.

The search strategy was developed in collaboration with a medical librarian specializing in systematic review searches, incorporating relevant free-text terms and medical subject headings related to the keywords “wound healing” and “*Astragalus mongholicus* Bunge”. A detailed search strategy is outlined in Supplementary Table S2, using PubMed as an example. This strategy was subsequently adapted for the other electronic databases to account for differences in syntax and controlled vocabulary. All searches were updated and rerun prior to the final analysis to incorporate the most recent eligible studies.

Besides, the researchers checked all references of the included articles and relevant reviews to identify any potentially overlooked studies that might not have been captured during the initial database searches.

### Selection

First, all identified citations were consolidated in Endnote X9.3.3, and duplicates were subsequently removed. Then, two researchers independently screened the titles and abstracts of the citations to identify potentially relevant studies. To guarantee data accuracy, a cross-checking process was carried out, and disagreements were resolved through discussion. After the initial screening, we retrieved the full texts of these potentially relevant citations as PDFs, assigned them unique identifiers, and randomly distributed them among the four independent researchers (working in two teams). The final selection of citations was conducted by consensus among the researchers of both groups.

### Data collection

Two reviewers independently extracted data from the included studies using a standardized data extraction form in Excel, and the third author checked the extracted data for accuracy. The extracted general data included study characteristics (e.g., the author’s name, publication year, and country of study), study populations (e.g., species, strain, gender, age, body weight, wound model, and wound creation method), intervention characteristics (e.g., the subtype of intervention, source, extraction method, dosage, dosing frequency, route of administration, and whether it was used alone or combined with novel materials), and comparators.

For primary and secondary outcomes, the number of animals in the intervention and control groups, the testing methods employed, and the mean values of each outcome with their standard deviation (sd) were extracted. When numerical data were not explicitly provided in the text, Web Plot Digitizer was used for data extraction from figures. All data extracted from graphs (e.g., bar graphs, line graphs) were cross-verified for clarity and accuracy. The sd value must be greater than zero, and the data reported as the standard error of the mean were converted to sd for the meta-analysis.

### Risk of bias assessment

The Systematic Review Centre for Laboratory Animal Experimentation (SYRCLE) risk of bias tool was used to assess each included study’s risk of bias (Hooijmans et al., 2014). Two researchers independently evaluated each study across the ten key domains outlined in the SYRCLE tool: sequence generation, baseline characteristics, allocation concealment, random housing, blinding of caregivers and investigators, random outcome assessment, blinding of outcome assessors, incomplete outcome data, selective outcome reporting, and other source(s) of bias. The potential risk of bias for each study was classified as high risk, unclear risk, or low risk. In cases where discrepancies arose between the two researchers regarding the risk of bias ratings, consensus was obtained by consulting a third reviewer.

### Data analysis

We performed a meta-analysis only for outcomes reported by two or more studies using the same measurement method. Since all outcomes were continuous and the studies exhibited variability in baseline wound sizes and measurement scales, we consequently chose the Hedges’g version of the standardized mean difference (SMD) with 95% confidence interval (CI) as the pooled effect size. This adjustment was applied to correct for potential overestimation bias inherent in the classic SMD when sample sizes are small. We assessed heterogeneity with the I^2^ statistic and chi-square test but employed a random-effects model for all pooled analyses. This decision was based on the expectation of substantial clinical and methodological heterogeneity among the studies. Studies that did not provide adequate data for meta-analysis were described narratively. Furthermore, if the results continuously increase or decrease at multiple time points, only the last time point will be selected for analysis. Similarly, for studies testing multiple effective doses, we used the dose associated with the maximum effect estimate in the pooled analysis. Subgroup analyses were performed based on disease models (e.g., chronic wounds vs. non-chronic wounds) and intervention characteristics (e.g., intervention subtype and combination with novel materials).

To test the robustness of the findings, a sensitivity analysis was conducted by iteratively excluding each study one at a time to assess its impact on the pooled results. Publication bias was evaluated using funnel plots and Egger’s test for outcomes including more than ten studies. Asymmetry in the funnel plot or a significant Egger’s test (*P* ≤ 0.05) was considered suggestive of potential publication bias. In such cases, the trim-and-fill method was employed to adjust for the bias and estimate its impact on the effect size.

All statistical analyses were performed using R software, with statistical significance set at α = 0.05.

## Results

### Study selection process

**FIGURE 1:**
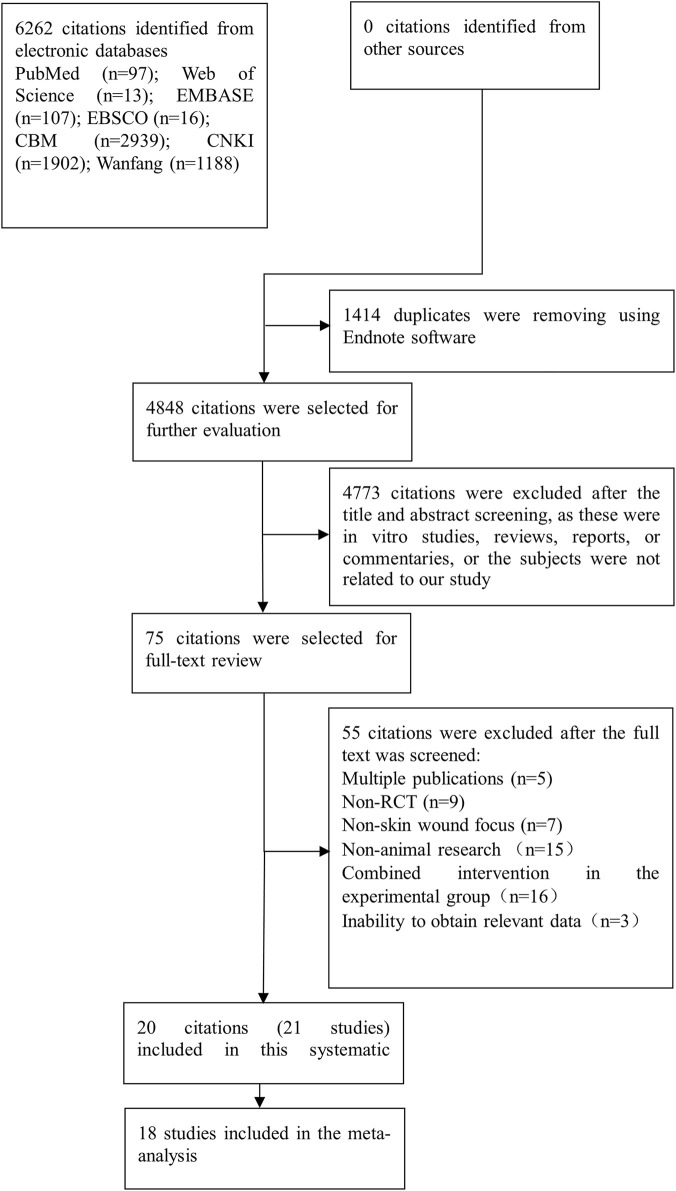
PRISMA flow chart of study selection.

The PRISMA flow diagram (Figure 1) depicts the literature selection process. Through a systematic search of the specified databases, a total of 6,262 citations were initially identified. After removing 1414 duplicates and screening the titles and abstracts, 75 full-text articles were retrieved and underwent full review according to the inclusion and exclusion criteria. Ultimately, 20 eligible citations (21 studies) were included in the systematic review (Cui et al., 2024; Du et al., 2024; Fan et al., 2022; Fan et al., 2024; Jia et al., 2024; Lau et al., 2008; Liang, 2015; Liu et al., 2024; Luo et al., 2016; Qiu, 2019; Shan, 2015; Wang et al., 2021; Wang et al., 2024; Xiong et al., 2024; Yang et al., 2015; Yue et al., 2022; Zhang, 2020; Zhang Z. et al., 2024; Zhao et al., 2017; Zheng et al., 2023), among which 18 were incorporated into the meta-analysis. The main reasons for excluding the remaining 55 articles are as follows: duplicate reporting of the same study, non-skin wound focus, non-animal research, combined intervention in the experimental group, inability to obtain relevant data, and non-random design.

### Study characteristics

The characteristics of the included studies are summarized in Table 1. All of these studies were conducted in China and published between 2008 and 2024. A total of 559 animals were incorporated into these 21 studies. The majority of the studies utilized rats (n = 17, 80.95%) (Cui et al., 2024; Du et al., 2024; Fan et al., 2022; Fan et al., 2024; Jia et al., 2024; Lau et al., 2008; Liu et al., 2024; Qiu, 2019; Wang et al., 2021; Wang et al., 2024; Xiong et al., 2024; Yue et al., 2022; Zhang, 2020; Zhang Z. et al., 2024; Zheng et al., 2023), primarily Sprague-Dawley (SD) rats (n = 15, 71.43%) (Cui et al., 2024; Du et al., 2024; Fan et al., 2022; Jia et al., 2024; Liu et al., 2024; Shan, 2015; Wang et al., 2021; Wang et al., 2024; Xiong et al., 2024; Yang et al., 2015; Yue et al., 2022; Zhang, 2020; Zhang Z. et al., 2024; Zheng et al., 2023), with the remainder using Wistar rats (n = 3, 14.29%) (Fan et al., 2024; Lau et al., 2008; Qiu, 2019). Mice were used in 14.29% (n = 3) (Liang, 2015; Luo et al., 2016; Zhao et al., 2017) of the studies, specifically the C57BL/6 strain (n = 2, 9.52%) (Luo et al., 2016; Zhao et al., 2017) and the BALB/c strain (n = 1, 4.76%) (Liang, 2015). Male animals were the dominant gender used in the research (n = 15, 71.43%) (Cui et al., 2024; Du et al., 2024; Jia et al., 2024; Lau et al., 2008; Liang, 2015; Liu et al., 2024; Luo et al., 2016; Qiu, 2019; Wang et al., 2024; Xiong et al., 2024; Yang et al., 2015; Zhang, 2020; Zhang Z. et al., 2024; Zhao et al., 2017; Zheng et al., 2023). In 14.29% (n = 3) (Shan, 2015; Wang et al., 2021) of the studies, only female animals were used, and another 9.52% (n = 2) (Fan et al., 2024; Fan et al., 2022) used animals of both sexes. One study (4.76%) did not report the sex of the animals (Yue et al., 2022). The age of the animals spanned from 6 to 12 weeks; however, this information was not reported in seven of the studies (Du et al., 2024; Jia et al., 2024; Liang, 2015; Liu et al., 2024; Qiu, 2019; Shan, 2015; Zhao et al., 2017).

**TABLE 1:** Characteristics of the included studies.

Study	Country	Animals (species, strain, gender, age)	Animal disease model	Number of animals in case VS control groups	Wound model	Intervention	Route of administration	Follow up period	Control
Cui et al. (2024)	China	Rats, SD, male, 8–10 weeks	-	6 VS 6	Full-thickness skin excision wound	AS-IV	Topical administration	8 days	Normal saline
Fan et al. (2024)	China	Rats, Wistar, male and female, 11–12 weeks	Diabetes	30 VS 30	Full-thickness skin excision wound	AS-IV	Topical administration	14 days	Normal saline
Zhang Z. et al. (2024)	China	Rats, SD, male, 6–8 weeks	Diabetes	10 VS 10	Full-thickness skin excision wound	APS	Intraperitoneal injection	10 days	Normal saline
Jia et al. (2024)	China	Rats, SD, male, NR	Diabetes	12 VS 13	Full-thickness skin excision wound	*Astragalus mongholicus* Bunge	Topical administration	14 days	Normal saline
Liu et al. (2024)	China	Rats, SD, male, NR	Diabetes	12 VS 12	Full-thickness skin excision wound	AS-IV loaded dressing	Topical administration	15 days	Blank gauze
Xiong et al. (2024)	China	Rats, SD, male, 6 weeks	Diabetes	5 VS 5	Full-thickness skin excision wound	AS-IV loaded dressing	Topical administration	14 days	Blank gauze
Wang et al. (2024)	China	Rats, SD, male, 8 weeks	Diabetes	12 VS 12	Full-thickness skin excision wound	AS-IV loaded dressing	Topical administration	14 days	Blank gauze
Du et al. (2024)	China	Rats, SD, male, NR	-	10 VS 10	Full-thickness skin excision wound	APS loaded dressing	Topical administration	14 days	Blank gauze
Zheng et al. (2023)	China	Rats, SD, male, 7 weeks	-	10 VS 10	Deep partial-thickness burn wounds	APS	Intraperitoneal injection	7 days	Normal saline
Fan et al. (2022)	China	Rats, SD, male and female, adults	-	10 VS 10	Full-thickness skin excision wound	APS	Topical administration	21 days	PBS
Yue et al. (2022)	China	Rats, SD, NR, 8 weeks	Diabetes	4 VS 4	Full-thickness skin excision wound	APS loaded dressing	Topical administration	15 days	Blank gauze
Wang et al. (2021)	China	Rats, SD, female, 8 weeks	Diabetes	10 VS 10	Full-thickness skin excision wound	AS-IV	Intraperitoneal injection	15 days	Normal saline
Zhang (2020)	China	Rats, SD, male, 12 weeks	Diabetes	10 VS 10	Full-thickness skin excision wound	APS	Intragastric administration	14 days	Normal saline
Qiu (2019)	China	Rats, Wistar, male, NR	Diabetes	32 VS 32	Full-thickness skin excision wound	*Astragalus mongholicus* Bunge	Intragastric administration	21 days	Normal saline
Zhao et al. (2017)	China	Mice, C57BL/6, male, NR	-	13 VS 13	Full-thickness skin excision wound	APS	Topical administration	21 days	PBS
Luo et al. (2016)	China	Mice, C57BL/6, male, 8–10 weeks	Diabetes	10 VS 10	Full-thickness skin excision wound	AS-IV	Topical administration	23 days	DMSO
Shan 2015–1	China	Rats, SD, female, NR	-	8 VS 8	Full-thickness skin excision wound	AS-IV	Topical administration	21 days	Normal saline
Shan 2015–2	China	Rats, SD, female, 8 weeks	-	8 VS 8	Burn wounds	AS-IV	Topical administration	31 days	Normal saline
Liang (2015)	China	Mice, BALB/c, male, NR	-	27 VS 27	Full-thickness skin excision wound	APS	Intragastric administration	8 days	PBS
Yang et al. (2015)	China	Rats, SD, male, 10 weeks	Diabetes	6 VS 6	Full-thickness skin excision wound	APS loaded dressing	Topical administration	20 days	Blank gauze
Lau et al. (2008)	China	Rats, Wistar, male, 8 weeks	Diabetes	35 VS 33	Full-thickness skin excision wound	*Astragalus mongholicus* Bunge	Intragastric administration	18 days	Water

Abbreviation: SD, Sprague-Dawley; AS-IV, Astragaloside IV; APS, astragalus polysaccharides; NR, not report.

Regarding wound types, chronic and non-chronic wounds were investigated in 71.43% (n = 15) (Cui et al., 2024; Fan et al., 2022; Fan et al., 2024; Jia et al., 2024; Lau et al., 2008; Liu et al., 2024; Luo et al., 2016; Qiu, 2019; Wang et al., 2021; Wang et al., 2024; Xiong et al., 2024; Yang et al., 2015; Yue et al., 2022; Zhang, 2020; Zhang Z. et al., 2024) and 28.57% (n = 6) (Du et al., 2024; Liang, 2015; Shan, 2015; Zhao et al., 2017; Zheng et al., 2023) of the studies, respectively. Among the studies focusing on chronic wounds, the majority (n = 13, 61.91%) adopted a streptozotocin induced diabetic model (Fan et al., 2024; Jia et al., 2024; Lau et al., 2008; Liu et al., 2024; Luo et al., 2016; Qiu, 2019; Wang et al., 2021; Wang et al., 2024; Xiong et al., 2024; Yang et al., 2015; Yue et al., 2022; Zhang, 2020; Zhang Z. et al., 2024). Moreover, full-thickness excisional wounds emerged as the most commonly used wound model, accounting for 85.71% (n = 18) (Cui et al., 2024; Du et al., 2024; Fan et al., 2022; Fan et al., 2024; Jia et al., 2024; Lau et al., 2008; Liang, 2015; Liu et al., 2024; Luo et al., 2016; Qiu, 2019; Wang et al., 2021; Zhang J. et al., 2024; Xiong et al., 2024; Yang et al., 2015; Yue et al., 2022; Zhang, 2020; Zhang Z. et al., 2024; Zhao et al., 2017) of the studies. These wounds were primarily located on the dorsal skin (n = 16, 76.19%) (Cui et al., 2024; Du et al., 2024; Fan et al., 2022; Fan et al., 2024; Liang, 2015; Liu et al., 2024; Luo et al., 2016; Qiu, 2019; Wang et al., 2021; Wang et al., 2024; Xiong et al., 2024; Yang et al., 2015; Yue et al., 2022; Zhang, 2020; Zhang Z. et al., 2024; Zhao et al., 2017) and the dorsum of the foot (n = 2, 9.52%) (Jia et al., 2024; Lau et al., 2008). Of these, the majority (n = 15, 71.43%) (Cui et al., 2024; Fan et al., 2022; Fan et al., 2024; Lau et al., 2008; Liang, 2015; Liu et al., 2024; Luo et al., 2016; Qiu, 2019; Wang et al., 2021; Wang et al., 2024; Yang et al., 2015; Yue et al., 2022; Zhang, 2020; Zhang Z. et al., 2024; Zhao et al., 2017) were designed in a circular shape, with wound diameters varying from 5 to 20 mm.

As presented in Table 1, among the 21 studies, three (14.29%) investigated *Astragalus mongholicus* Bunge (Jia et al., 2024; Lau et al., 2008; Qiu, 2019), nine (42.86%) focused on AS-IV (Cui et al., 2024; Fan et al., 2024; Liu et al., 2024; Luo et al., 2016; Shan, 2015; Wang et al., 2021; Wang et al., 2024; Xiong et al., 2024), and nine (42.86%) examined APS (Du et al., 2024; Fan et al., 2022; Liang, 2015; Yang et al., 2015; Yue et al., 2022; Zhang, 2020; Zhang Z. et al., 2024; Zhao et al., 2017; Zheng et al., 2023). Among the three studies investigating *Astragalus mongholicus* Bunge, two (Jia et al., 2024; Qiu, 2019) used commercially available Astragalus injection (a sterile aqueous preparation for clinical use), and one (Lau et al., 2008) used a self-prepared aqueous extract. Of the nine studies that examined AS-IV, eight employed commercially obtained AS-IV monomer, and only one (Fan et al., 2024) used AS-IV-enriched extracts prepared by ethanol extraction (with details of extraction methods provided in Supplementary Table S3). For the nine studies focusing on APS, four (Fan et al., 2022; Zhang Z. et al., 2024; Zhang, 2020; Zheng et al., 2023) utilized commercially purchased APS monomer, three (Yang et al., 2015; Yue et al., 2022; Zhao et al., 2017) used aqueous extracts prepared in-house (with details of extraction methods provided in Supplementary Table S3), and two (Du et al., 2024; Liang, 2015) did not report the source in sufficient detail.

The most commonly used route of administration was local administration (dressing/smearing) (n = 14, 66.67%) (Cui et al., 2024; Du et al., 2024; Fan et al., 2022; Fan et al., 2024; Jia et al., 2024; Liu et al., 2024; Luo et al., 2016; Shan, 2015; Wang et al., 2024; Xiong et al., 2024; Yang et al., 2015; Yue et al., 2022; Zhao et al., 2017), which was either mixed with a vehicle (n = 7, 33.33%) or embedded in hydrogels or other scaffolds (n = 6, 28.57%). Notably, in one study (Fan et al., 2024), AS-IV powder was directly applied to cover the wound. In addition, intragastric (n = 4, 19.05%) (Lau et al., 2008; Qiu, 2019; Liang, 2015; Zhang, 2020) and intraperitoneal (n = 3, 14.29%) (Wang et al., 2021; Zhang Z. et al., 2024; Zheng et al., 2023) routes were frequently employed. Seventeen studies reported the administration dosage (Cui et al., 2024; Du et al., 2024; Fan et al., 2022; Jia et al., 2024; Lau et al., 2008; Liang, 2015; Luo et al., 2016; Qiu, 2019; Shan, 2015; Wang et al., 2021; Wang et al., 2024; Xiong et al., 2024; Yang et al., 2015; Zhang, 2020; Zhang Z. et al., 2024; Zhao et al., 2017; Zheng et al., 2023), four (Lau et al., 2008; Liang, 2015; Wang et al., 2021; Zheng et al., 2023) of which took the size of the animals into account. The dose exhibited significant heterogeneity across the studies. Three studies evaluated dose-response with three gradient doses (Qiu, 2019; Zhang Z. et al., 2024; Zhang, 2020), and one study evaluated low and high doses (Cui et al., 2024). In these studies, wound healing was reported to be positively correlated with the dosage. Additionally, the follow-up periods varied, mostly ranging from 8 to 28 days. For more details, please refer to Supplementary Table S3.

### Result of risk of bias assessment

**FIGURE 2:**
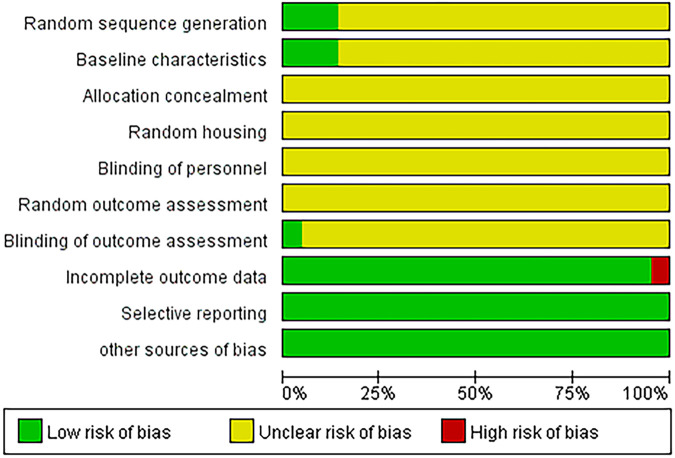
Quality assessment of the included studies.

We assessed the risk of bias in the included animal studies using the SYRCLE’s risk of bias tool. Overall, most of the elements investigated were judged as having an unclear risk of bias. Although all studies mentioned randomization, only three (Du et al., 2024; Fan et al., 2024; Zheng et al., 2023) explicitly described the method used for generating the random sequence. Regarding baseline characteristics, while detailed animal data were provided in all studies, only three (Fan et al., 2024; Lau et al., 2008; Qiu, 2019) reported that baseline characteristics were comparable between the control and experimental groups after allocation. All studies were rated as having an unclear risk of bias in allocation concealment, random housing, blinding during intervention, and random outcome assessment, as no relevant details were reported. For blinding of outcome assessors, only one study (Luo et al., 2016) stated that histological evaluations were performed by blinded assessors. One study (Jia et al., 2024) was judged to have a high risk of attrition bias, while the remaining studies were rated as low risk. In terms of selective reporting, all studies were considered low risk based on the methods described, although none had a publicly available pre-published protocol to confirm this assessment. Similarly, other potential sources of bias were generally evaluated as low risk. For details, please refer to Figure 2 and Supplementary Table S4.

### Result of meta-analysis

All 21 included studies examined the effect of *Astragalus mongholicus* Bunge or its primary metabolites (for convenience’s sake, these interventions are hereafter collectively referred to as *Astragalus mongholicus* Bunge) on wound healing. Overall, in preclinical studies, the application of *Astragalus mongholicus* Bunge to *in vivo* wound models significantly stimulated angiogenesis, enhanced collagen deposition, reduced the inflammatory response, and ultimately accelerated wound healing. The detailed results are presented below.

### Wound healing

**FIGURE 3:**
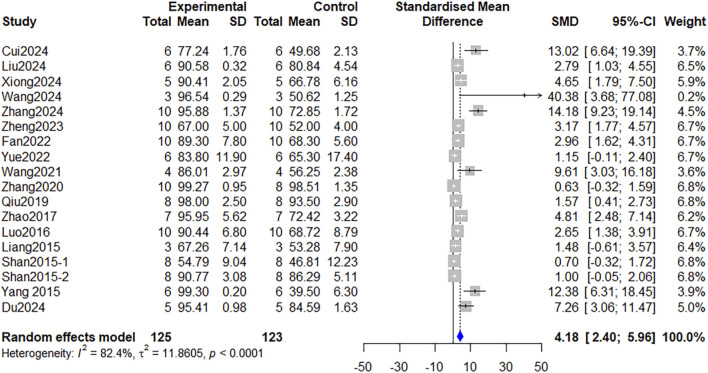
Forest plot comparing the wound contraction percentage between the *Astragalus mongholicus* Bunge group and the control group.

Wound healing, as the primary outcome, was measured by all 21 included studies in the form of either the wound contraction percentage or the precise time to complete wound closure. In 20 studies that used the percentage of wound contraction, the data from Fan et al. (Fan et al., 2024) and Lau et al. (Lau et al., 2008) were unavailable in a format suitable for pooling with other datasets, as these two studies used the absolute healed wound area as the primary outcome measure. Therefore, data from 18 studies (Cui et al., 2024; Du et al., 2024; Fan et al., 2022; Jia et al., 2024; Liang, 2015; Liu et al., 2024; Luo et al., 2016; Qiu, 2019; Shan, 2015; Wang et al., 2021; Wang et al., 2024; Xiong et al., 2024; Yue et al., 2022; Zhang, 2020; Zhang Z. et al., 2024; Zhao et al., 2017; Zheng et al., 2023) involving 248 animals were included in the meta-analysis focusing on the wound contraction percentage. As shown in Figure 3, compared with the control group, the administration of *Astragalus mongholicus* Bunge significantly promoted wound healing [SMD = 4.18, 95%CI: 2.40–5.96, *P* < 0.0001]. The findings of Fan et al. (Fan et al., 2024) and Lau et al. (Lau et al., 2008) also corroborated this conclusion.

**FIGURE 4:**
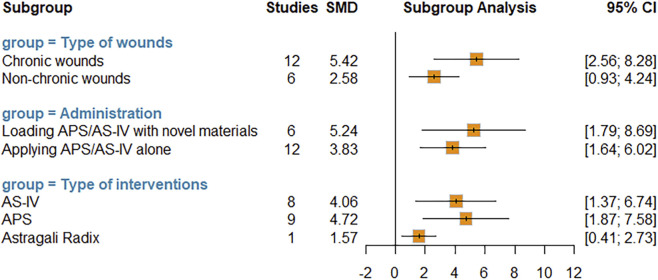
Subgroup analysis demonstrating the effect of wound types (chronic wounds vs. non-chronic wounds), administration route (loading APS/AS-IV with novel materials VS appling APS/AS-IV alone), and intervention subtypes (AS-IV, APS, and *Astragalus mongholicus* Bunge) on the wound contraction percentage.

Furthermore, given the presence of high heterogeneity (I^2^ = 82%), we performed a subgroup analysis for this primary outcome to explore the potential sources of heterogeneity and identify factors influencing therapeutic efficacy, based on the following categories: wound types, intervention subtypes, and combination with novel materials. The subgroup analysis results indicated that heterogeneity remained high across all subgroups (Supplementary Table S5), reflecting the variability in wound preparation, dosage regimen, and outcome measurement. Additionally, no significant differences in effect sizes were observed between different wound types (*P* = 0.09), intervention subtypes (*P* = 0.05), or the presence/absence of combination with novel materials (*P* = 0.50) (Figure 4, Supplementary Table S4).

Notably, we found that the reporting of precise time to complete wound closure was frequently overlooked in the included studies, with only 2 studies (Jia et al., 2024; Yang et al., 2015) reporting specific time points for wound closure. In Jia et al.’ s study, the results revealed that animals assigned to the *Astragalus mongholicus* Bunge aqueous extract group exhibited a shortened healing time in comparison to control condition. Similarly, in the study by Yang et al., wounds in the APS-loaded fiber group healed significantly faster than those in the model group and the blank fiber grou.

### Angiogenesis

Angiogenesis is crucial for wound healing, as new blood vessels deliver oxygen and nutrients to the repairing tissue. To evaluate this process, the included studies frequently measured key markers of vascularization, including vascular endothelial growth factor (VEGF), platelet endothelial cell adhesion molecule-1 (CD31), and α-smooth muscle actin (α-SMA). Across all studies that assessed these markers, treatment with *Astragalus mongholicus* Bunge consistently demonstrated beneficial effects on promoting angiogenesis.

#### VEGF

**FIGURE 5:**
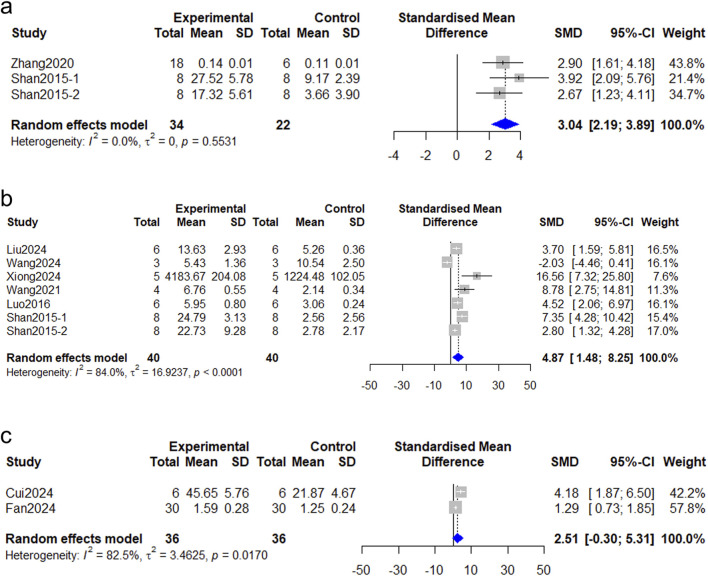
Forest plot comparing the angiogenesis between the *Astragalus mongholicus* Bunge group and the control group. **(a)** VEGF; **(b)** CD31; **(c)** α-SMA.

Seven studies (Du et al., 2024; Fan et al., 2022; Jia et al., 2024; Luo et al., 2016; Shan, 2015; Zhang, 2020) reported the assessment of vascularization degree via VEGF, all of which demonstrated benefit with the use of *Astragalus mongholicus* Bunge. However, only three (Shan, 2015; Zhang, 2020) of these studies provided eligible data for meta-analysis, as all three employed the same detection technique, immunohistochemistry, to assess VEGF expression in wounds. As depicted in Figure 5a, there was no heterogeneity among these studies (I^2^ = 0%, *P* > 0.05). The pooled data demonstrated a significantly higher VEGF expression in wound tissue of the intervention group (SMD = 3.04, 95%CI: 2.19–3.89, *P* < 0.0001).

This finding is corroborated by results from other detection techniques. Three additional studies, utilizing immunofluorescence, Western blot (WB), and enzyme-linked immunosorbent assay (ELISA) on wound tissues, serum, or dorsal pedis arteries, respectively, consistently confirmed the upregulating effect of *Astragalus mongholicus* Bunge on VEGF expression (Du et al., 2024; Fan et al., 2022; Jia et al., 2024).

Furthermore, evidence at the transcriptional level was provided by a separate study, which used quantitative real-time polymerase chain reaction (qRT-PCR) to quantify VEGF messenger ribonucleic acid (mRNA) in treated tissues and demonstrated that AS-IV significantly upregulated its expression (Luo et al., 2016).

#### CD31

Seven studies (Liu et al., 2024; Luo et al., 2016; Shan, 2015; Wang et al., 2024; Xiong et al., 2024) investigated the expression levels of CD31 in wounds, which is a key marker of neovascularization. The pooled analysis demonstrated that *Astragalus mongholicus* Bunge increased the expression of CD31 after treatment compared to controls (SMD = 4.87, 95%CI: 1.48–8.25, *P* = 0.0048) (Figure 5B). The heterogeneity index was high (I^2^ = 84%).

#### α-SMA

Two studies (Cui et al., 2024; Fan et al., 2024) detected mature blood vessels using α-SMA specific immunofluorescence staining. As Figure 5C showed, the meta-analysis results demonstrate no significant difference in α-SMA staining of wound tissue between rats treated with AS-IV and control rats [SMD = 2.51, 95% CI: 0.30–5.31, *P* = 0.08, I^2^ = 82.5%].

#### von Willebrand factor (vWF)

Two studies (Luo et al., 2016; Xiong et al., 2024) reported assessments of vWF. However, data pooling was not feasible due to heterogeneity in measurement techniques. In Luo’s study, qRT-PCR showed increased vWF expression in AS-IV treated wounds compared with wounds without positive treatment (Luo et al., 2016). Accordingly, in the other study, the levels of this proangiogenic factor in the skin tissue of wound rats incubated with AS-IV intervention were also increased (Xiong et al., 2024).

### Collagen deposition

**FIGURE 6:**
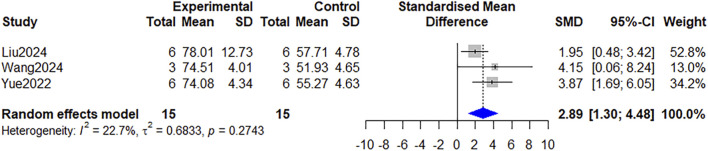
Forest plot comparing the collagen deposition between the *Astragalus mongholicus* Bunge group and the control group.

Collagen deposition, another pivotal determinant of effective wound healing, was examined in 9 studies (Cui et al., 2024; Fan et al., 2024; Liu et al., 2024; Luo et al., 2016; Shan, 2015; Wang et al., 2024; Yang et al., 2015; Yue et al., 2022). Across them, 6 studies evaluated total collagen deposition, organization, and maturation using Masson’s trichrome staining (Liu et al., 2024; Wang et al., 2024; Yue et al., 2022; Luo et al., 2016; Shan, 2015). All of these six studies reported there was an increased deposition of collagen fibers in wounds treated with *Astragalus mongholicus* Bunge when compared to the control group. Furthermore, three studies (Liu et al., 2024; Wang et al., 2024; Yue et al., 2022) provided specific quantitative data eligible for meta-analysis. The pooled results demonstrated a statistically significant increase in collagen deposition in the *Astragalus mongholicus* Bunge group (SMD = 2.89, 95% CI: 1.30 to 4.48, *P* = 0.0004) (Figure 6).

Additionally, at the protein level, three studies (Cui et al., 2024; Fan et al., 2024; Yang et al., 2015) quantified the expression of type I collagen in treated tissues *via* immunofluorescence and SR staining. All of them showed that animals treated with *Astragalus mongholicus* Bunge exhibited higher levels of type I collagen than those without treatment. Similarly, in the study by Luo et al., an early upregulation of collagen-related genes, including collagen llla and fibronectin, was reported in *Astragalus mongholicus* Bunge treated wounds compared with the control group (Luo et al., 2016). Meanwhile, Yang et al. characterized the dynamic expression profile of type III collagen, noting an initial peak followed by a gradual decline until the study endpoint (Yang et al., 2015).

### Inflammation

In most included studies, wound tissue samples were usually used for histological assessment of the infiltration by inflammatory cells and quantitation of inflammatory factors at the protein level or the transcriptional level. Furthermore, two studies (Fan et al., 2022; Jia et al., 2024) utilized blood samples to measure C-reactive protein (CRP) levels or used ELISA to quantitate levels of inflammatory factors, aiming to evaluate the regulatory effect of *Astragalus mongholicus* Bunge on systemic inflammation. Overall, *Astragalus mongholicus* Bunge exerts a powerful anti-inflammatory effect, significantly lowering the levels of pro-inflammatory cytokines.

#### Histological assessment

Hematoxylin-eosin staining analysis of wound tissues revealed that the *Astragalus mongholicus* Bunge treated groups exhibited significantly reduced infiltration of inflammatory cells, predominantly macrophages and neutrophils. As the experiment progressed, the extent of inflammatory cell infiltration in the *Astragalus mongholicus* Bunge groups continued to decrease, and by the endpoint across the six studies (Cui et al., 2024; Liu et al., 2024; Shan, 2015; Wang et al., 2021; Yue et al., 2022), these inflammatory cells were scarcely detectable in the wound tissue. In contrast, the control groups consistently displayed marked inflammatory cell infiltration, indicative of a persistent inflammatory response.

#### Tumour necrosis factor-α (TNF-α)

**FIGURE 7:**
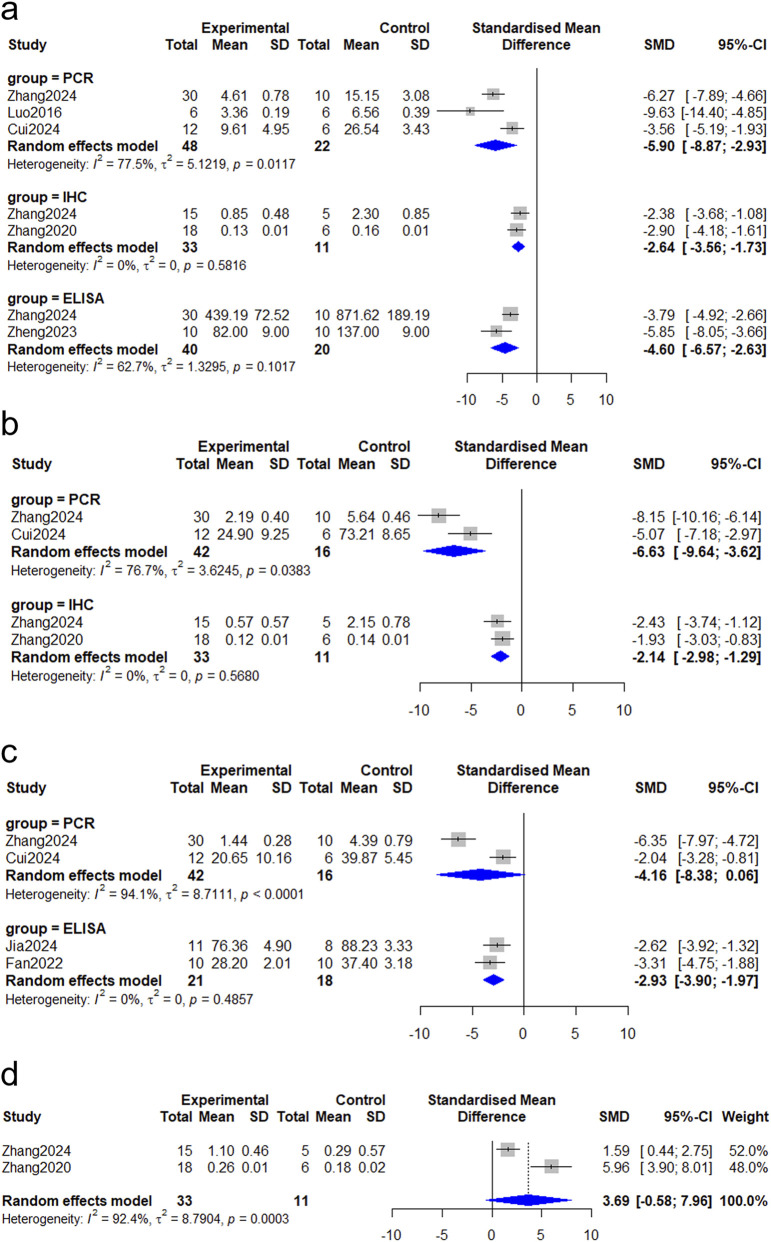
Forest plot comparing inflammatory factors between the *Astragalus mongholicus* Bunge group and the control group. **(a)** TNF-α; **(b)** IL-1β; **(c)** IL-6; **(d)** IL-10.

Five studies reported on TNF-α (Cui et al., 2024; Fan et al., 2024; Luo et al., 2016; Zhang, 2020; Zheng et al., 2023). Our meta-analysis demonstrates that TNF-α levels in *Astragalus mongholicus* Bunge treated wounds were significantly lower than in controls, irrespective of the detection method (immunohistochemistry: SMD = −2.64, 95% CI: 3.56 to −1.73, *P* < 0.0001; ELISA: SMD = −4.60, 95% CI: 6.57 to −2.63, *P* < 0.0001) (Figure 7a). This reduction at the protein level is further supported by evidence of TNF-α mRNA downregulation in wound tissue (SMD = −5.90, 95% CI: 8.87 to −2.93, *P* < 0.0001). Corroborating this finding, Zheng et al. independently observed a decrease in TNF-α mRNA in the blood samples of *Astragalus mongholicus* Bunge treated animals (Zheng et al., 2023).

#### Interleukin-1β (IL-1β)

Three studies (Cui et al., 2024; Fan et al., 2024; Zhang, 2020) investigated IL-1β levels in wounds with the administration of *Astragalus mongholicus* Bunge. As shown in Figure 7b, a significant downregulation of IL-1β was observed in *Astragalus mongholicus* Bunge treated wounds at both the protein (SMD = −2.14, 95% CI: 2.98 to −1.29, *P* < 0.0001) and mRNA levels (SMD = −6.63, 95% CI: 9.64 to −3.62, *P* < 0.0001).

#### Interleukin-6 (IL-6)

Two studies (Fan et al., 2022; Jia et al., 2024) were included in the analysis of the effects of intervention on IL-6 at the protein levels. The pooled data revealed significantly lower IL-6 expression in wounds treated with *Astragalus mongholicus* Bunge (SMD = −2.93, 95% CI: 3.90 to −1.97, *P* < 0.0001) (Figure 7C). However, the pooled analysis from another two studies (Cui et al., 2024; Fan et al., 2024) showed that there was no significant difference in IL-6 mRNA between the two groups (SMD = −4.16, 95% CI: 8.38 to 0.06, P = 0.0531) (Figure 7c). It should be noted that these two analyses are based on different types of samples, one being wound tissue and the other being blood.

#### Interleukin-10 (IL-10)

Two studies (Zhang Z. et al., 2024; Zhang, 2020) investigated the impact of *Astragalus mongholicus* Bunge on IL-10 expression in wound tissue samples using immunohistochemistry (IHC). The pooled analysis indicated that *Astragalus mongholicus* Bunge tended to promote the upregulation of this anti-inflammatory cytokine; however, the difference between the two groups was not statistically significant (SMD = 3.69, 95% CI: 0.58 to 7.96, *P* = 0.0905) (Figure 7d).

#### Macrophage polarization

Macrophages were investigated in three studies (Cui et al., 2024; Luo et al., 2016; Zhang, 2020). Given the heterogeneity in the macrophage subtypes examined across the three studies and the lack of specific quantitative data in some reports, we omitted the pooled estimate and only performed a narrative synthesis.

In Luo’s study, the result revealed that the number of F4/80+CD206+ cells was significantly higher in AS-IV-treated mice compared with vehicle-treated mice (Luo et al., 2016). In particular, this study also reported upregulated expression of genes associated with M2 macrophages, including arginase-1, Ym1, and IL-13 expression in treated wounds (Luo et al., 2016). Similarly, in the study by Zhang et al., although no quantitative data were provided, immunofluorescence results indicated enhanced M2 macrophage polarization in wounds following the administration of APS (Zhang, 2020). Furthermore, Cui et al. determined the presence of mRNA encoding inducible nitric oxide synthase (iNOS) in injured tissues by qRT-PCR, and a notable reduction was observed in wounds treated with AS-IV (Cui et al., 2024).

#### Other inflammatory factors

Two studies explored other inflammatory factors. In Zheng et al.’ study, the expression levels of IL-2 and interferon-γ in wound tissues and serum were assessed (Zheng et al., 2023). The results demonstrated that APS can alleviate systemic and local inflammation by activating the ILK/AKT/GSK-3β signaling pathway. In another study by Jia et al., the aqueous extract of *Astragalus mongholicus* Bunge was also proven to modulate systemic inflammation levels, as the levels of CRP in the group treated with this extract were significantly lower than those in the model control group (Jia et al., 2024).

These results suggest that *Astragalus mongholicus* Bunge plays a critical role in dampening inflammatory pathways, which may have significant implications for therapeutic strategies in tissue repair.

### Growth factors

**FIGURE 8:**
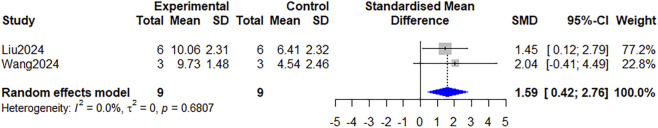
Forest plot comparing the TGF-β level between the *Astragalus mongholicus* Bunge group and the control group.

The effects of *Astragalus mongholicus* Bunge on key growth factors were evaluated in five studies. A pooled analysis of two studies (Liu et al., 2024; Wang et al., 2024) demonstrated that *Astragalus mongholicus* Bunge enhances the expression of transforming growth factor-β (TGF-β) in wounds after treatment compared to controls (SMD = 1.58, 95% CI: 0.42 to 2.76, *P* = 0.0078, I^2^ = 0%) (Figure 8). Additionally, one study suggested that *Astragalus mongholicus* Bunge may also induce the expression of epidermal growth factor (EGF) and basic fibroblast growth factor (bFGF) during wound healing (Zhao et al., 2017). Not surprisingly, another study confirmed the upregulated mRNA expression profiles encoding these proteins in groups receiving *Astragalus mongholicus* Bunge treat ment (Zheng et al., 2023).

### Adverse events

No harmful events were reported in any of the included studies. In the study by Jia et al., five rats died in the model control group and one died in the *Astragalus mongholicus* Bunge aqueous extract group (Page et al., 2021). However, the cause of death was not specified.

### Publication bias

**FIGURE 9:**
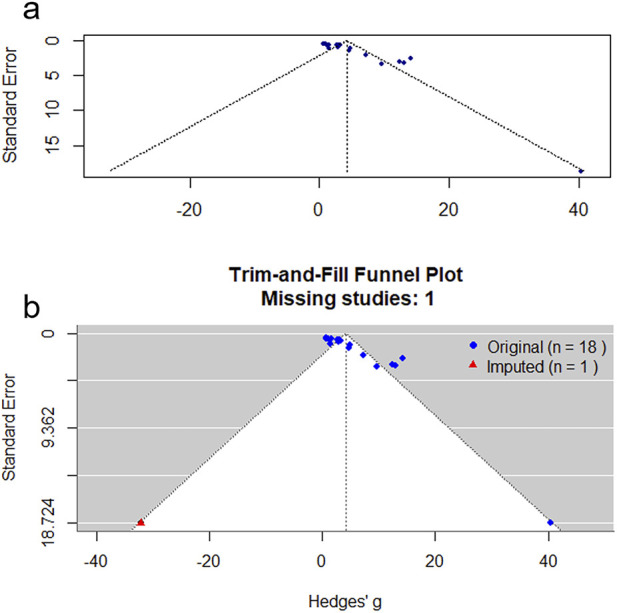
Funnel plot **(a)** and trim-and–fill analysis publication bias **(b)**.

We constructed a funnel plot based on the primary outcome measure—the percentage of wound contraction—to assess for publication bias. This was because only in this analysis did the number of included studies exceed 10. The funnel plot displayed asymmetry, with studies distributed asymmetrically around the funnel plot’s symmetry line, suggesting the possible presence of publication bias (Figure 9a). The result of Egger’s test was t = 7.33, *P* < 0.0001, which confirmed this asymmetry. Therefore, we employed the trim-and-fill method to evaluate the potential impact of publication bias on the initial conclusion.

Through this analysis, only one theoretically missing experiment was denoted by a red triangle (Figure 9b). The recalculated summary analysis results demonstrate sustained consistency without any instances of reversal, thereby indicating the robustness and stability of our analysis findings (pre-trim-and-fill: SMD = 4.18, 95%CI: 2.40–5.96, *P* < 0.0001; post-trim-and-fill: SMD = 4.10, 95%CI: 2.32–5.87, *P* < 0.0001).

### Sensitivity analysis

**FIGURE 10:**
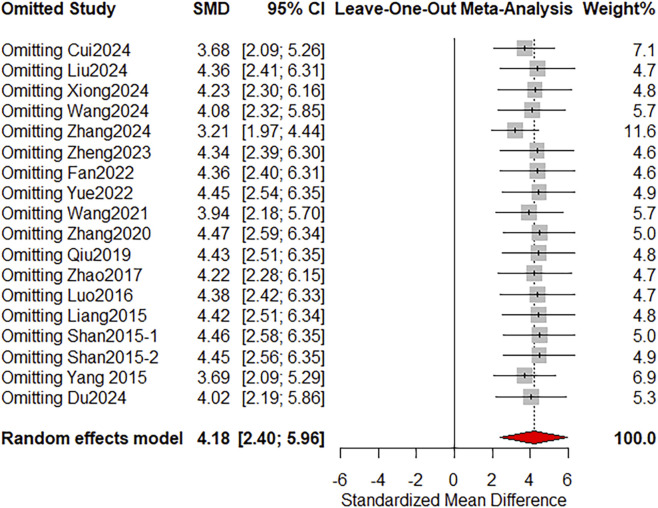
Sensitivity analysis of the wound contraction percentage.

We conducted a sensitivity analysis using the “leave-one-out” method for the primary outcome indicator. This was done to assess the influence of individual studies on the SMD as the effect-size metric. According to Figure 10, the newly synthesized results were consistent with the original results, indicating the high dependability of our meta-analysis.

## Discussion

### General findings

This systematic review aimed to evaluate the therapeutic efficacy and safety of *Astragalus mongholicus* Bunge in preclinical wound models. Consistent with this objective, all 21 included studies employed *in vivo* wound models, which are well-established and relevant for investigating the complex, multi-stage process of wound healing. Our synthesis of these studies revealed that *Astragalus mongholicus* Bunge significantly promoted wound healing. Furthermore, no adverse effects were reported in these included studies, supporting the safety of this intervention for promoting wound healing and skin regeneration. Subgroup analysis suggested that differences in the active composition of intervention, combination with novel materials, or wound models among studies had no significant effect on the efficacy of treatment.

Furthermore, to explore the possible mechanisms through which *Astragalus mongholicus* Bunge promotes tissue regeneration, its effects on specific micro-processes were investigated. Generally, this botanical drug is primarily thought to facilitate healing by stimulating angiogenesis, enhancing collagen synthesis, and modulating inflammatory responses.

As we know, normal angiogenesis is indispensable for wound closure, as it delivers essential oxygen, nutrients, and immune cells to the wound site, all of which are crucial for cellular proliferation, migration, and tissue repair. Impaired or absent angiogenesis is a hallmark of delayed wound healing in humans (Veith et al., 2019).

VEGF is one of the most potent pro-angiogenic cytokines present in the skin, promoting the proliferation, migration, and tube formation of endothelial cells (Nissen et al., 1998). Consistent with previous reports, our meta-analysis results indicates that *Astragalus mongholicus* Bunge can upregulate VEGF expression in wound areas.

Additionally, in numerous studies, the percentage of CD31 or α-SMA positive cells was usually utilized as an indicator to assess angiogenesis in the wound bed. CD31 is a transmembrane protein closely associated with newly formed blood vessels. The presence of CD31-positive cells indicates the existence and activity of endothelial cells (Kim et al., 2009). And that α-SMA is predominantly expressed by vascular smooth muscle cells, which contribute to the stabilization of nascent blood vessels through mural cell recruitment and extracellular matrix synthesis (McAndrews et al., 2022). Thus, these two markers reflects not only the density of newly formed vessels but also their maturity and functional competence. Our meta-analysis revealed that CD31 expression levels were significantly higher in wounds treated with *Astragalus mongholicus* Bunge. Although no statistically significant effects were observed from pooling data on α-SMA, discussion of some trends was possible. As expected, there was a tendency toward upregulation of α-SMA expression. Therefore, *Astragalus mongholicus* Bunge enhances not only the formation but also the maturation of functional blood vessels, thereby improving the wound microenvironment and accelerating healing. However, among the studies included in our meta-analysis, only a limited number further explored the underlying mechanism through which *Astragalus mongholicus* Bunge promotes angiogenesis. According to these studies, *Astragalus mongholicus* Bunge may upregulate hypoxia-inducible factor-1α (HIF-1α) and VEGF expression *via* activation of the PI3K/AKT signaling pathway, thus facilitating wound angiogenesis (Cui et al., 2024; Fan et al., 2022; Jia et al., 2024).

Moreover, our study confirmed that *Astragalus mongholicus* Bunge treatment significantly enhances collagen deposition in the wound bed—another pivotal determinant of effective wound healing. Collagen, the primary component of the extracellular matrix, plays multifaceted and indispensable roles in wound repair (Gardeazabal and Izeta, 2024). In this systematic review, included studies demonstrated through histological and quantitative assessments that *Astragalus mongholicus* Bunge can promote the deposition of key extracellular matrix proteins, including collagen I, collagen III, and fibronectin. Among these, three studies quantitatively reported collagen deposition rates, and our pooled analysis supports this conclusion. However, it should be noted that excessive collagen accumulation may lead to adverse outcomes such as keloid formation (Gardeazabal and Izeta, 2024). Most of the included studies focused primarily on the early and middle stages of wound healing, with limited data available on the remodeling and scarring phase. Consequently, current evidence remains insufficient to fully evaluate the long-term effects of *Astragalus mongholicus* Bunge on collagen maturation and scar formation.

Another crucial factor is the inflammatory response. Acute inflammatory response caused by tissue injury is the primary stage of injury repair (Sorg and Sorg, 2023). It secretes a variety of cytokines related to wound healing and activates immune cells, including macrophage cells and neutrophil cells, to eliminate pathogens and necrotic tissues and promote the healing of wounds (Sorg and Sorg, 2023). However, previous studies have shown that persistent and severe inflammation often delayed wound healing, since inflammatory cells will release excessive amounts of proteolytic enzymes and reactive oxygen species (ROS), which can further destroy the newborn extracellular matrix in the wound area and cause additional damage to newborn cells (Landén et al., 2016).

A growing number of studies show that *Astragalus mongholicus* Bunge has a major anti-inflammatory function. Similarly, the results of our meta-analysis clearly show that treatment with *Astragalus mongholicus* Bunge leads to a significant reduction in the levels of TNF-α, IL-1β, and IL-6, which are prominent pro-inflammatory factors. Furthermore, the findings of Luo et al. and Cui et al. indicate that *Astragalus mongholicus* Bunge may promote macrophage transition from the M1 to the M2 phenotype by activating the SIRT1/NF-κB pathway, which subsequently suppresses iNOS and enhances arginase-I activity (Cui et al., 2024; Luo et al., 2016). It is well known that the phenotypic transformation of macrophages from a proinflammatory M1 to an anti-inflammatory M2 state constitutes a pivotal factor influencing various cellular functions such as proliferation, motility, and vascularization (Willenborg and Eming, 2014). Overall, *Astragalus mongholicus* Bunge exhibits immunomodulatory-promoting characteristics, which play a role in facilitating wound healing.

Collectively, the mechanisms discussed above—angiogenesis, collagen deposition, and inflammation modulation—provide a robust explanation for the therapeutic efficacy of *Astragalus mongholicus* Bunge in wound healing. Moreover, these effects may be further amplified by the antioxidant properties of *Astragalus mongholicus* Bunge. This antioxidant property, widely documented for *Astragalus mongholicus* Bunge and other medical natural products (Selamoglu et al., 2015; Shahzad et al., 2016), helps mitigate oxidative damage and create a regenerative microenvironment conducive to tissue repair.

Beyond its efficacy, the favorable safety profile, wide availability, and low cost of *Astragalus mongholicus* Bunge further support its potential as a practical and sustainable therapeutic option for wound care, particularly in resource-limited settings. However, the administration strategy of *Astragalus mongholicus* Bunge for wound treatment is a key point that needs to be focused on in future studies. Among the included studies, *Astragalus mongholicus* Bunge was administered *via* various routes, such as intraperitoneal injection, intragastric administration, and topical application. However, for wound treatment, topical administration is more clinically acceptable and rational. Nevertheless, both *Astragalus mongholicus* Bunge itself and its two primary active metabolites face inherent limitations, such as unstable physicochemical properties, limited bioactive absorption, and low bioavailability, which hinder their effective clinical translation when applied directly to wounds. Thus, developing a safe and efficient drug delivery system is essential to enhance the bioavailability of this botanical drug. Studies have shown that incorporating an active ingredient into novel materials can increase the hydrophilicity and stability of the substance and enable sustained and stable release, thereby enhancing the bioavailability (Alberti et al., 2017; Qi et al., 2022).

In six (Du et al., 2024; Jia et al., 2024; Liu et al., 2024; Wang et al., 2024; Xiong et al., 2024; Zhang J. et al., 2024) of the included studies, *Astragalus mongholicus* Bunge has been combined with novel materials, most of which are based on nanofiber dressing and hydrogels. Subgroup analysis showed that there was no statistically significant difference in wound healing efficacy between *Astragalus mongholicus* Bunge used in combination with these materials and *Astragalus mongholicus* Bunge used alone. This means that the composite formulations can achieve the same therapeutic effect as the original drug. Differently, some recent meta-analyses showed that utilizing novel materials in conjunction with active ingredients could more effectively enhance collagen deposition, angiogenesis, and wound healing (Al-Masawa et al., 2022; Aghayan et al., 2025). We thought that the limited number of studies available for subgroup analysis in this meta-analysis may have reduced statistical power, potentially explaining the lack of observed efficacy differences.

Regrettably, due to the small number of studies and heterogeneity, we were unable to conduct a direct analysis comparing the combination of *Astragalus mongholicus* Bunge with materials to the use of *Astragalus mongholicus* Bunge alone. Such an analysis could further substantiate the synergistic benefits and enhance the relevance of our findings. So, future studies should prioritize direct comparisons of combination *versus* single-component interventions in standardized preclinical models.

### Limitation

As is common in systematic reviews and meta-analyses, this study has several notable limitations.

First and foremost, substantial heterogeneity was observed across the included studies. Particularly, the 21 included studies collectively reported almost 40 different outcome measures, a variability compounded by differences in measurement methods and assessment timepoints even for the same outcome. This inconsistency in outcome reporting and measurement compromises the ability to accurately synthesize and compare data, thereby reducing the overall utility and reliability of the findings.

Furthermore, the absence of standardized criteria for evaluating treatment efficacy led to the omission of several clinically relevant indicators. For example, although the timing of wound closure is a critical endpoint, only two studies (Jia et al., 2024; Yang et al., 2015) reported the specific time to complete wound healing—an outcome of direct importance to clinical trial design and application.

As is well recognized in preclinical research, studies investigating similar interventions for the same condition often employ divergent outcome measures. In fact, inconsistency and a lack of standardization in the selection and measurement of outcomes are pervasive not only in preclinical studies but also in clinical trials (Zhang Z. et al., 2024). To address this issue in clinical research, the Core Outcome Set (COS) framework has been developed (Williamson et al., 2017). We contend that it is equally critical and timely to establish standardized outcome sets for preclinical studies.

Furthermore, substantial variability was also observed across studies in terms of animal models, wound types, and intervention protocols, leading to a high degree of heterogeneity. Combining such diverse comparisons in a single meta-analysis may have artifactually reduced the observed statistical heterogeneity, thereby compromising the reliability of the comparative findings.

Secondly, the inadequate reporting of the included studies hindered a thorough assessment of bias risks. Results from the SYRCLE risk of bias tool indicated that most studies were judged to have an unclear risk of bias, which may lead to overestimation or underestimation of the pooled effects in our meta-analysis. Thus, it could compromise the reliability of the research conclusions and limit their clinical translational significance. Preclinical studies that minimize the biases present more solid scientific results. Therefore, we emphasize the importance of authors adhering to the Animal Research: Reporting of *In Vivo* Experiments (ARRIVE) guidelines (Percie, et al., 2020), which facilitate transparent assessment of bias risk and enhance the reproducibility of experimental findings.

Thirdly, in order to avoid ignoring relevant studies, many measures were taken, including performing the search in the most important databases in the field of health sciences, formulating the sensitive search strategy by several researchers, manually searching the references of included studies, and following the double independent review process. However, the evaluation results of publication bias for the primary outcome indicated that publication bias existed in this study.

Nevertheless, despite existing heterogeneity and certain risks of bias across the included studies, *Astragalus mongholicus* Bunge demonstrated consistent positive efficacy, supporting its potential for clinical translation.

## Conclusion

In conclusion, this review and meta-analysis demonstrates that *Astragalus mongholicus* Bunge significantly enhances wound closure, collagen deposition, and angiogenesis while modulating inflammatory cytokines, highlighting the huge potential of this medicinal plant to be used for wound treatments. However, translating these findings into clinical practice is challenged by high study heterogeneity, including variations in animal models, intervention strategy, and outcome measures. Such inconsistencies limit the practical applicability of preclinical results. Thus, more studies with comparable study protocols should be performed to validate the results of the present systematic review and meta-analysis. These future studies should leverage insights gained from our analysis, such as reducing potential risks of bias and aligning outcome reporting to propel studies towards definitive preclinical and initial clinical studies. Although there is a lengthy and challenging path from experimental animal models to clinical patient applications, we think that these challenges can be overcome in future research.

## Data Availability

The original contributions presented in the study are included in the article/Supplementary Material, further inquiries can be directed to the corresponding authors.

## Glossary

SYRCLE: the Systematic Review Centre for Laboratory Animal Experimentation
SMD: standardized mean difference
CI: confidence interval
SR: systematic review
APS: Astragalus polysaccharides
AS-IV: Astragaloside IV
PROSPERO: the International Prospective Register of Systematic Reviews
PRISMA: the Preferred Reporting Items for Systematic Reviews and Meta-Analyses
CBM: Chinese Biomedical Literature Database
CNKI: Chinese Digital Journals Full-text Database
SD: Sprague-Dawley
VEGF: vascular endothelial growth factor
CD31: platelet endothelial cell adhesion molecule-1
α-SMA: α-smooth muscle actin
WB: Western blot
ELISA: enzyme-linked immunosorbent assay
qRT-PCR: quantitative real-time polymerase chain reaction
mRNA: messenger ribonucleic acid
vWF: von Willebrand Factor
CRP: C-reactive protein
TNF-α: tumour necrosis factor-α
IL-1β: interleukin-1β
IL-6: interleukin-6
IL-10: interleukin-10
IHC: immunohistochemistry
iNOS: inducible nitric oxide synthase
TGF-β: transforming growth factor-β
EGF: epidermal growth factor
bFGF: basic fibroblast growth factor
HIF-1α: hypoxia-inducible factor-1α
ROS: reactive oxygen species
COS: the Core Outcome Set
ARRIVE: the Animal Research: Reporting of *In Vivo* Experiments.
