# Supplementary material for: Does Astragalus mongholicus Bunge help promote the healing of wounds? A systematic review and meta-analysis of preclinical animal studies
Source: Front Pharmacol. 2026 Apr 17;17:1799944. doi: 10.3389/fphar.2026.1799944 (PMC13132865; doi:10.3389/fphar.2026.1799944)
Supplement: Supplementary file 3 [file Table2.docx]

**Searching strategies: take PubMed for example**

#1 “Huang Qi” [Supplementary Concept]

#2 “Astragalus propinquus” [Mesh]

#3 astragaloside[Title/Abstract] OR astragalus[Title/Abstract] OR huangqi[Title/Abstract] OR huang qi[Title/Abstract] OR astragali radix[Title/Abstract] OR radix astragali[Title/Abstract]

#4 #1 OR #2 OR #3

#5 “Wound Healing” [MeSH]

#6 “Pressure Ulcer” [MeSH]

#7 “Leg Ulcer” [MeSH]

#8 chronic [Title/Abstract] AND (wound* [Title/Abstract] or ulcer*[Title/Abstract])

#9 bed sore*[Title/Abstract OR bedsore*[Title/Abstract] OR pressure sore*[Title/Abstract] OR pressure ulcer*[Title/Abstract] OR decubitus ulcer*[Title/Abstract] OR decubitus sore*[Title/Abstract] OR pressure injur*[Title/Abstract]

#10 foot ulcer*[Title/Abstract] OR plantar ulcer*[Title/Abstract] OR diabetic foot[Title/Abstract] OR diabetic feet[Title/Abstract]

#11 leg ulcer*[Title/Abstract] OR varicose ulcer*[Title/Abstract] OR venous ulcer*[Title/Abstract] OR stasis ulcer*[Title/Abstract] OR ulcus cruris[Title/Abstract] OR crural ulcer*[Title/Abstract] OR arterial

#12 diabetic[Title/Abstractj AND (wound*[Title/Abstract] ORulcer*[Title/Abstract])

#13 #5 OR #6 OR #7 OR #8 OR #9 OR #10 OR #11 OR #12

#14 #4 AND #13
