# Supplementary material for: Does Astragalus mongholicus Bunge help promote the healing of wounds? A systematic review and meta-analysis of preclinical animal studies
Source: Front Pharmacol. 2026 Apr 17;17:1799944. doi: 10.3389/fphar.2026.1799944 (PMC13132865; doi:10.3389/fphar.2026.1799944)
Supplement: Supplementary file 4 [file Table5.docx]

**The results of subgroup analyses for the wound contraction percentage**


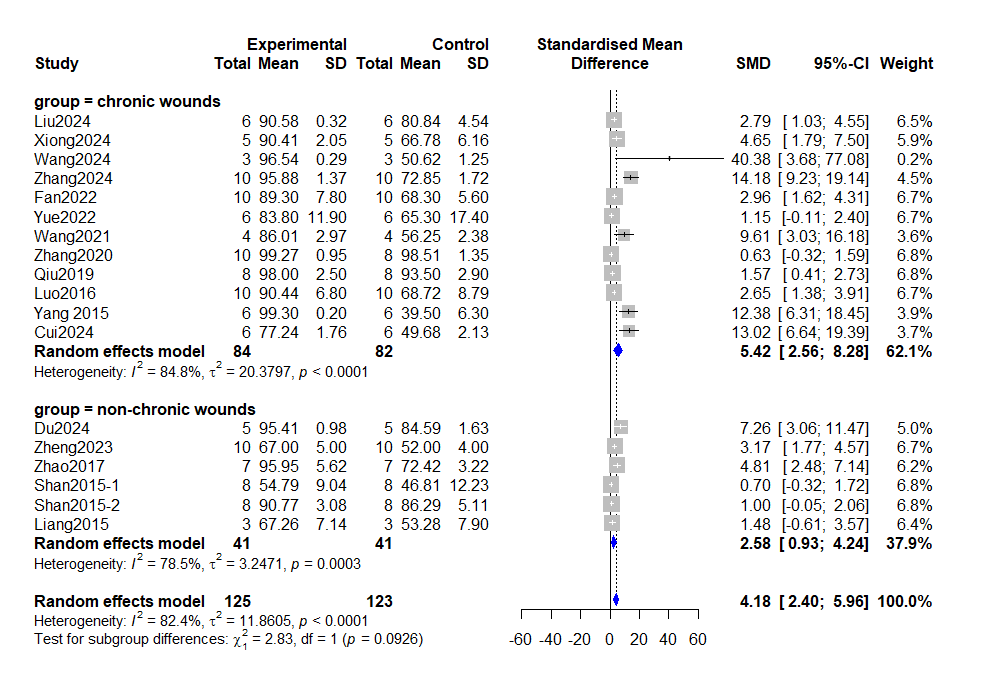


**Forest plot comparing the wound contraction percentage in chronic and non-chronic wounds between the *Astragalus mongholicus* Bunge group and the control group**


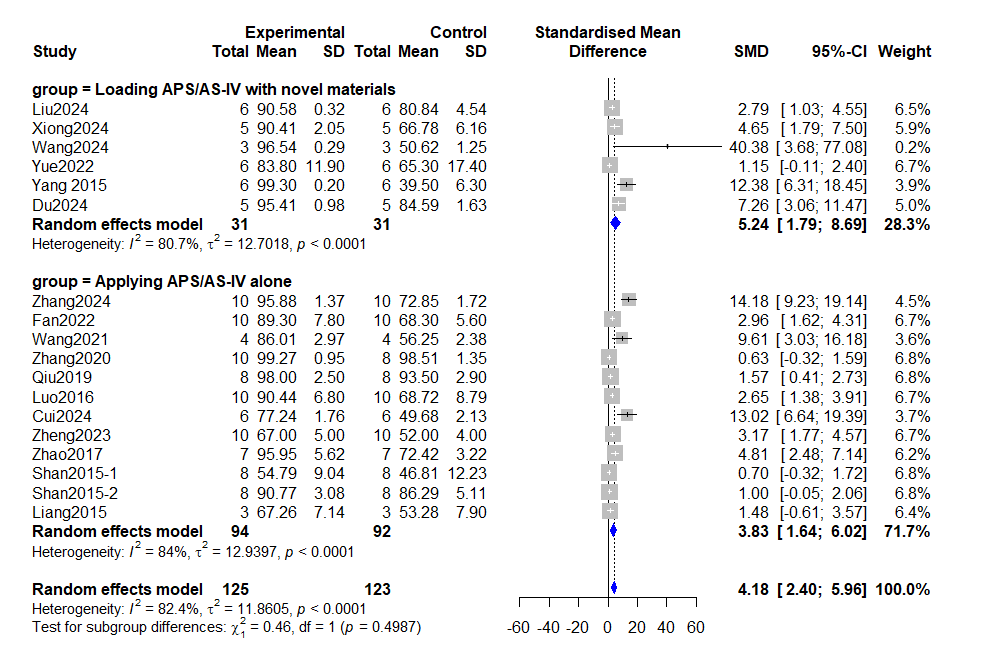


**Forest plot of the wound contraction percentage in wounds treated with *Astragalus mongholicus* Bunge, administered either in combination with or without novel materials**


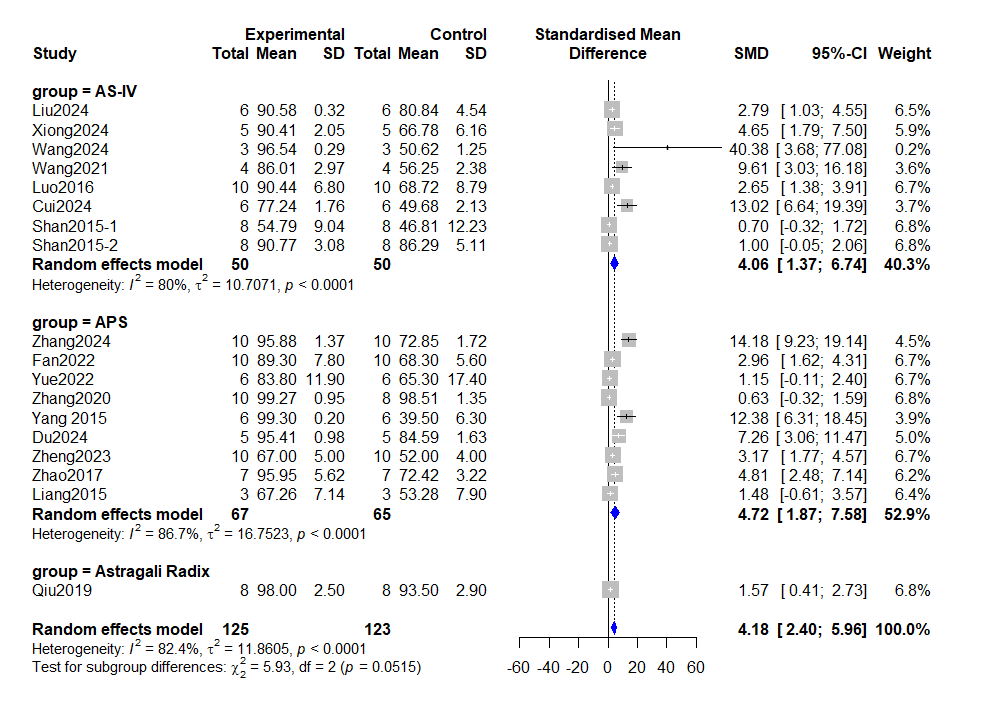
**Forest plot of the effect of intervention subtypes (AS-IV, APS, and *Astragalus mongholicus* Bunge) on wound contraction percentage**
