# Supplementary figures and images for: Does Astragalus mongholicus Bunge help promote the healing of wounds? A systematic review and meta-analysis of preclinical animal studies
Source: Front Pharmacol. 2026 Apr 17;17:1799944. doi: 10.3389/fphar.2026.1799944 (PMC13132865; doi:10.3389/fphar.2026.1799944)

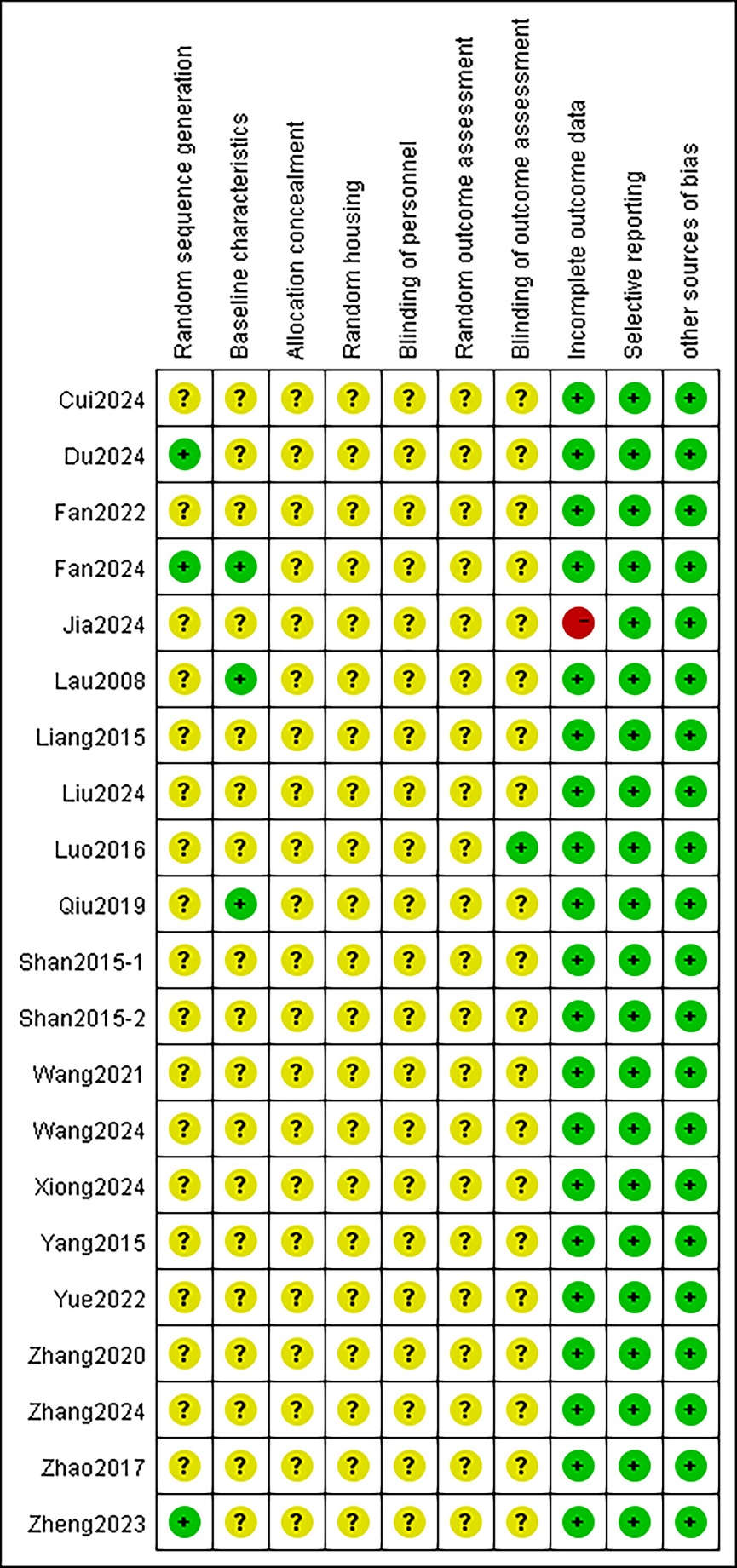


Quality assessment of individual studies

Supplement: Supplementary file 5 [file Table4.docx]
